# Performance of adolescents in oral narrative discourse and associated factors

**DOI:** 10.1590/2317-1782/20212021207

**Published:** 2022-04-15

**Authors:** Samantha Gomes Araújo, Vanessa de Oliveira Martins-Reis, Erica de Araújo Brandão Couto, Luciana Mendonça Alves

**Affiliations:** 1 Universidade Federal de Minas Gerais – UFMG - Belo Horizonte (MG), Brasil.; 2 Universidade de Brasília – UnB - Brasília (DF), Brasil.

**Keywords:** Narrative Discourse, Adolescent, Language Tests, Language Development, Picture Naming

## Abstract

**Purpose:**

to characterize the performance in oral narrative discourse of adolescents from 6 to 9 years of age from an elementary school, as well as to verify the influence of gender, school year, age, performance in oral language tests, memory, and appointment of figures.

**Methods:**

100 adolescents of both genders from the sixth to the ninth years of elementary school who did not have any language or learning disorders were evaluated for oral narrative discourse (MAC Battery), visual figure nomination (Boston Naming Test), oral language and memory (NEUPSILIN). Performance was considered as a response variable in narrative discourse (partial and complete retelling and the ability to answer questions). After univariate analysis, Multiple Linear Regression models were constructed.

**Results:**

Only general performance in the naming task was considered as a predictor of performance in oral narrative discourse. A direct association between the variables of narrative discourse and the naming of figures was present. We show the characteristics of adolescents' performance in the partial and complete retelling and in the answers to the questions by age, school year and sex.

**Conclusion:**

in the studied sample the participants (aged from two to seven years old) were able to understand and detail an oral narrative discourse similar to adults with a low educational level, consequently the MAC Battery narrative discourse test can be used to assess adolescents without any requirements for adaptation.

## INTRODUCTION

Adolescence is the period in which a young person develops from a child to an adult, ranging from ten to nineteen years of age. Linguistic competence in this age group is similar to that of adults^(1)^, with evolution of abstract language, comprehension and use of language^(2)^. Linguistic growth occurs during childhood, adolescence and adulthood and does not have any obvious completion point, therefore it is a dynamic and constantly expanding system^(3)^.

One of the skills of oral language is narrative discourse, which is defined as the linguistic ordering of temporally related events and actions^(4)^. This skill is essential for the development of written narrative^(5)^, for socio-emotional well-being and school performance^(6)^. Oral narrative discourse is an essential linguistic skill in communicative ability during adolescence. In social and academic use narrative discourse must be developed and requires the ability to remember events, organize information, understand the epistemological and emotional perspectives of others, employ complex syntax and appropriate vocabulary to express oneself clearly and accurately^(7)^.

Knowledge is limited about the linguistic and cognitive abilities of adolescents who principally speak Brazilian Portuguese both in clinical practice and in specific studies, consequently assessment tools and intervention strategies are scarce^(8)^. The lack of standardized tests with versions that are adapted and standardized for the adolescent population limits the achievement of objective data in the assessment of language of this age group.

A way to cater for the limited amount of assessment instruments specifically available for adolescents is to adapt validated and recognized tests for adults whose purpose is to assess linguistic and cognitive functions. Standardized instruments for the processing of oral narrative discourse of Brazilian Portuguese speaking adolescents were not found.

### Characterization of oral narrative discourse

The overall structure of the narrative is usually characterized by an initial configuration, complicating actions and resolution, which are hierarchically organized into three segments beginning, middle and end^(9)^.

Oral narrative ability can be assessed in two different ways, comprehension and production and is composed of macrostructure and microstructure levels. The macrostructure level relates to the components between sentences (cohesion, coherence, formulation of a mental model or the essence of a story) while the microstructure refers to the construction within the sentence (semantic, lexical, phonological and syntactic aspects of the emission)^(10)^.

A study carried out with bilingual children in the first and second years of school aimed to investigate the relationships between the micro and macro level domains of oral narratives within and between the English and Spanish languages through storytelling. The results showed that patterns of oral narrative development can be similar in both languages and a series of significant correlations within and between languages identified vocabulary as a significant predictor of macro-level speech scores in both languages^(11)^.

In the process of understanding an oral narrative, the ability to infer information that is not directly indicated in the story is influenced by the characteristics of the text, cognitive and contextual factors^(12)^. Studies suggest that the ability to make inferences contributes to language comprehension^(13)^. The questions to assess narrative comprehension are literal, inferential and aim to obtain information about the ability to understand sentences, as well as to establish relationships between ideas central to the theme of the narrative^(14)^.

Prosody is a relevant skill for processing the discursive structure, as it interacts and adds value to other language subsystems, such as syntax and semantics, facilitating language comprehension. Prosodic cues help to segment the speech flow into sentences, words and syllables, inform the syntactic structure and emphasize information aiding the understanding of the narrative discourse^(15)^. A study carried out with 79 students aged between 7 and 12 years of age from the second to the fifth grades investigated the prosodic aspects of oral language and reading comprehension and it is suggested that age and education would be related to the ability of listening comprehension. Younger and less educated children would be more dependent on prosodic factors, whilst older and more educated children would have more developed cognitive skills, which would enable them to understand a text regardless of prosodic variation^(16)^.

### Oral narrative discourse in adolescence

Oral narrative discourse reflects the level of cognitive development^(3)^ and the accomplishment of linguistic and mnemonic processing, among other underlying cognitive functions^(17)^, it is a determining skill in an adolescent's school and social life^(3)^. Adolescents can demonstrate great diversity in the use of complex syntax in oral narrative discourse^(18)^.

The learning process of oral narratives and its constituent elements, is gradual as the central nervous system develops^(19)^ so does the progression of reading. Educational levels^(20)^ and socioeconomic background are associated with language ability in adolescence^(21)^.

The mental organization in adolescents with language disorders is inadequate, resulting in problems with sequencing and structuring of a discourse, which compromises the ability to narrate events^(2)^. Language problems in adolescence is an area of interest for healthcare and education professionals as these disorders negatively impact academic performance, participation in the social sphere and professional guidance. To characterize adolescent language problems requires a good understanding of the typical development of language skills of this age group.

Another study carried out with adolescents with an average age of 14 years old who had typical language development aimed to create a new narrative task and to try to determine whether it would cause greater syntactic complexity than a conversational task. This research addressed the need for appropriate tools to assess the ability of oral narrative with syntactic complexity in eighth grade students. The results verified that there were no statistically significant differences in the performance of oral narrative between boys and girls and the narrative task raised greater syntactic complexity than the conversational task^(18)^.

The use of standardized language assessments makes it possible to measure linguistic characteristics and help to understand the acquisition process and contribute to the development of cognitive abilities and is also the basis for speech therapy intervention. The language of children and adults has been studied, but the transitional phase, I.e., that of adolescence is still relatively unexplored, especially the skills of oral narrative discourse. A better understanding of oral narrative discourse in adolescence can help to develop effective tools for intervention programs for this age group^(5)^. The aim of this study was to characterize the performance of adolescents, from the 6th to 9th grade of elementary school, in the production and understanding of an oral narrative discourse, as well as the influence of gender, school year, age, picture naming, general score of oral language and memory. In addition, we sought to verify whether the oral narrative discourse task of the Montreal Communication Assessment Battery – MAC Battery, validated and standardized for Brazilian Portuguese speaking adults can be used for the adolescent population without requiring any form of adaptation.

## METHODS

This is an analytical cross-sectional observational study, approved by the Research Ethics Committee of the Federal University of Minas Gerais number 1.722.230.

Adolescents eligible to participate in the study and their parents were informed about the voluntary aspects of the study, its benefits and its stages they then signed a consent form.

One hundred adolescents from two public schools in the same region of Belo Horizonte were included in the study. Participants did not have any previous diagnoses of learning disorders either sensory, neurological, cognitive or behavioral changes as confirmed by the schools coordinators and were native speakers of Brazilian Portuguese without any fluency in a second language. The schools already had a list of adolescents with neurodevelopmental disorders and from this list was possible to identify and exclude some of the participants. Adolescents identified by teachers and coordinators who had poor school performance were also excluded.

The age group of the participants ranged from 11 to 16 years of age, with an average age of 12.9 years (SD=1.2) and 62% of the participants were female. During the data collection period, the adolescents attended elementary school in the following school years: 6th grade (n=34), 7th grade (n=23), 8th grade (n=25) and 9th grade (n=18).

The instruments used for this study were as follows, the oral narrative discourse test of the Montreal Communication Assessment Battery (MAC Battery)^(17)^, Boston Naming Test (BNT)^(22)^ and oral language and memory tests of the Brief Neuropsychological Assessment Instrument (NEUPSILIN)^(23)^.

To assess the oral narrative discourse, the MAC Battery test was used, consisting of partial retelling of the story, paragraph by paragraph, full retelling of the story, tasks of giving a title and observation of inference processing (understanding the moral of the story)^(17)^. For usage and analysis, the procedures proposed by the authors of the tests in the instruction manual were followed. In the retelling tasks, the adolescents listened to a short text which they recounted in a summarized form and in their own words of what had just happened in the story, first in each paragraph (partial retelling). The cells corresponding to the total of essential information remembered (maximum score=18) and the total of present information remembered (maximum score=29) were registered. Then, the adolescent listened to the same story and were asked to recount the entire text (full retelling, maximum score=13). Deviant communicative behaviors were noted, such as: abundant personal observations, tangential discourse, non-respect to the timeline of events, omission of relationship markers, imprecise lexicon, imprecise references, addition of incorrect information and lack of fluency. Then the adolescent was asked to answer 12 questions about the story and, later given the option of changing the title of the story. Based on retelling tasks, answers to comprehension questions, and non-verbal language (such as laughs and personal comments), it was verified whether the inference of the narrative was made, that is, the understanding of the moral of the story^(17)^. The average time of application of the Oral Narrative Discourse task was 15 minutes.

The reading of the text for the task of retelling the story was standardized so that there was no difference in intonation and prosody that could interfere with the adolescent's understanding of the text. The text was recorded on a Sony WDC WD3200BEVT-75ZCT2 recorder by a theatrical actor with 12 years of experience.

Picture naming ability was assessed using the BNT. The pictures were presented in the order of the test, allowing twenty seconds for each answer. If the participant answered incorrectly or not at all the examiner provided a phonemic cue. If the participant continued to answer incorrectly or not at all within twenty seconds a semantic suggestion related to the picture already indicated on the answer sheet was provided. The transcription of the answers given by the participants was registered in the test registration protocol. For analysis of the test, the score corresponding to the adolescent's answers was analyzed, namely: total correct answers without a cue, total answer with phonemic cues (correct and incorrect), total answer with semantic cues (correct and incorrect), general total of correct answers (spontaneous evocation + evocation with cues)^(22)^. The average time of application of the Boston Naming Test was 20 minutes.

The total score of the NEUPSILIN oral language subtest^(24)^ was analyzed. The test presents normative values for the Brazilian Portuguese speaking population older than 12 years of age. The oral language subtest consists of naming, repetition, automatic language, comprehension, and inference processing tasks. One point was assigned for correct answers and zero for incorrect ones; for analysis, the general score corresponding to the adolescent's total score was added. Adolescent performance was classified as adequate and inadequate, considering the test reference. Adolescents' performance was recorded on a standard test answer sheet. On average the test lasted 7 minutes.

The participants also took the NEUPSILIN memory subtest^(23)^. Each test of the memory subtest was analyzed separately, consisting of tasks of working memory, episodic-semantic verbal memory, long-term semantic memory, and short-term visual memory. To analyze the results, a quantitative assessment of the adolescents' responses was made, corresponding to each task in the test. Student performance was classified as adequate and inadequate according to standardized test data. On average the test lasted 7 minutes.

Data collection was carried out between August 2016 and March 2017. The tests mentioned in this article were carried out separately, totaling three sessions with each participant. All assessments were carried out individually at the school, during lesson times in a room designated for this purpose, the application norms and standardized analyzes of the tests were carefully followed. The test sequence started with the NEUPSILIN^(23)^ global assessment of oral language and memory followed by the BNT^(22)^ and then the MAC Battery^(17)^.

The database was built using Excel and after consistency analysis, univariate descriptive analyzes were performed using the Statistical Software Package for Social Sciences (SPSS) version 19.0. Initially the Kolmogorov-Smirnov test was carried out to assess the normality of the continuous numerical variables and all of these showed a normal distribution. Descriptive analysis was performed by calculating frequencies and measures of central tendency and dispersion (average and standard deviation). Simple Student t tests were carried out to compare two independent means and Anova to compare three independent means and finally Pearson correlation to relate the two continuous numeric variables.

For the analyses, the adolescents' performance in the comprehension and expression of oral discourse the response variable, and explanatory variables, gender, school year, age, total correct answers in the Boston Naming Test and global oral language score (naming, repetition, automatic language, comprehension, and inference processing) and memory (working memory, verbal episodic-semantic and prospective) in the NEUPSILIN test.

Finally multiple linear regression models were built with narrative discourse as the outcome (partial and full retelling), and as explanatory variables all those that presented p value less than 0.20 in the univariate analysis. The backwards method was adopted, removing the variables with the highest p-value until the final adjusted model was obtained.

## RESULTS

The results section will be presented by topic. Initially, the results of descriptive, univariate, and multivariate analysis of the partial and full retelling will be presented. In the second topic the results of the association between the variables of the retelling task of the MAC Battery and the variables of the Boston Naming Test will be presented, followed by a specific topic for marks on the closed questions.

### Partial and full recounts and associated factors

Table 1 shows the results of the descriptive statistics of the adolescents' narrative discourse. Concerning the title, initially only 30% gave titles that demonstrated that the inference was made. Most adolescents (79%) kept the title and made inferences about the moral of the story (77%). The moment of inference that prevailed was after the first hearing (42%).

**Table 1:** Descriptive Statistics: Narrative Discourse of Adolescents

Variable		Frequency (n)	Prevalence (%)
Copious personal observations			
yes		2	2.0
no		98	98.0
Tangential Discourse			
yes		2	2.0
no		98	98.0
Failure to respect the timeline of events			
yes		21	21.0
no		79	79.0
Omission of relationship markers			
yes		0	0.0
no		100	100.0
Imprecise lexicon			
yes		5	5.0
no		95	95.0
Inaccurate references			
yes		6	6.0
no		94	94.0
Supplemented with incorrect information			
yes		15	15.0
no		85	85.0
Lack of fluency			
yes		1	1.0
no		99	99.0
Title given by adolescent			
Title that demonstrates that the inference was made		30	30.0
Title related to the story, but not indicative of inference processing		64	64.0
Title unrelated to the story or incorrect relationship		6	6.0
Student kept the title			
yes		79	79.0
no		21	21.0
Score for new title			
Title that demonstrates that the inference was made.		15	15.0
Title related to the story, but not indicative of inference processing.		6	6.0
Title unrelated to the story or incorrect relationship		2	2.0
Conserved title		77	77.0
Inference made			
yes		77	77.0
no		23	23.0
Time the inference was processed			
0- not present		24	24.0
1- During		2	2.0
2- After the first reading		42	42.0
3- During		3	3.0
4- after the second reading		24	24.0
5- in providing the first title		0	0.0
6 – on text comprehension issues		5	5.0
7 – in the provision of the second title		0	0.0
8 – another moment		0	0.0
Variable	**Total - test**	**Average**	**SD**
Total essential information remembered in partial retelling	18	12.6	3.3
Total present information remembered in partial retelling	29	16.0	4.6
Total of ideas remembered in the full retelling	13	9.8	2.5
Total correct answers to questions	12	9.6	3.0

SD = standard deviation

Of the six types of deviant communicative behavior in the narrative discourse, the adolescents presented non timeline of events (21%), incorrect information (15%), inaccurate references (6%) and inaccurate lexicon (5%).

Of a total of 18 pieces of essential information remembered the average of the adolescents' answers was 12.6 points and in 29 pieces of present information remembered the average was 16 points in the partial retelling of the story. Regarding the complete retelling of the story, in a total of 13 pieces of information remembered, the average of the adolescents' answers was 9.8 points. In the assessment of text comprehension, in a total of 12 questions, the average was 9.6 points (Table 1).

This study did not find any association between the performance of adolescents in the retellings of the narrative discourse test and the explanatory variables gender, education and adequacy in oral language, according to the results of univariate analyzes between performance in the narrative discourse test and the explanatory variables.

Table 2 shows the results of the Pearson correlation between performance in the partial and full retelling of the MAC Battery test and the variables age, memory, general performance in oral language and picture naming. There is a moderate direct correlation between the partial and full retelling and the total number of correct answers in the naming. In this case the better picture naming performance, the greater the total essential and present information remembered in the partial retelling and the total information remembered in the full retelling. The other variables were not associated with the partial and full retelling.

**Table 2:** Pearson's correlation results for the association between performance in the retelling of the MAC Battery narrative discourse test and the variables, age, memory, oral language and picture naming

Variable	Total essential information remembered in partial recount		Total present information remembered in partial recount		Total ideas remembered in full recount	
	**r**	**p value**	**r**	**p value**	**r**	**p value**
Age	0.126	0.212	0.122	0.227	0.128	0.206
Memory	0.142	0.166	0.160	0.120	0.024	0.817
working memory	0.101	0.326	0.122	0.238	-0.051	0.619
Digit ascending ordering	0.051	0.625	0.132	0.200	-0.029	0.777
Auditory span of words into sentences	0.122	0.277	0.121	0.242	-0.021	0.841
Episodic-semantic verbal memory	0.129	0.209	0.104	0.312	0.108	0.293
Immediate recall	0.072	0.485	0.030	0.771	-0.005	0.962
Late recall	0.179	0.082	0.161	0.118	0.122	0.082
Recognition	0.043	0.679	0.040	0.700	0.114	0.269
Long-term semantic memory	0.010	0.922	0.075	0.495	0.034	0.746
Short term visual memory	-0.020	0.893	0.072	0.486	-0.004	0.965
Oral language	-0.041	0.692	0.047	0.650	-0.083	0.424
Total correct answers (naming)	0.443	<0.001*	0.463	<0.001*	0.500	<0.001*

* Significant p≤0,05;

r = correlation coefficient

The performance in the Boston Naming Test remained in the three multiple linear regression models. According to the final models an increase of 1 point in the total correct answers in naming increases 0.322 points in the total of essential information remembered in the partial retelling, 0.225 point in the total of present information remembered in the partial retelling, and 0.192 point in the total of ideas remembered in the full retelling.

### Narrative discourse versus figure naming

As the overall performance in the Boston Naming Test was the only variable predicting the performance of adolescents in retelling, it was decided to verify the association of all test variables with the narrative discourse. Pearson Correlation results are shown in Table 3.

**Table 3:** Pearson correlations between performance in partial and full retelling and performance in naming with and without semantic and phonemic cues

Total essential information remembered in partial retelling	r	valor p
Total correct answers with no cue	0.461	<0.001*
Total correct answers with semantic cue	-0.225	0.033*
Total incorrect answer with semantic cue	-0.400	<0.001*
Total correct answer with phonemic cue	0.068	0.204
Total incorrect answer with phonemic cue	-0.479	<0.001*
Total correct answers	0.443	<0.001*
Total present information remembered: partial recount	**r**	**valor p**
Total correct answers with no cue	0.468	<0.001*
Total correct answers with semantic cue	-0.201	0.046*
Total incorrect answer with semantic cue	-0.394	<0.001*
Total correct answer with phonemic cue	0.092	0.364
Total incorrect answer with phonemic cue	-0.502	<0.001*
Total correct answers	0.463	<0.001*
Total of remembered ideas: full recount	**r**	**valor p**
Total correct answers with no cue	0.556	<0.001*
Total correct answers with semantic cue	-0.280	0.005*
Total incorrect answer with semantic cue	-0.495	<0.001*
Total correct answer with phonemic cue	0.018	0.856
Total incorrect answer with phonemic cue	-0.559	<0.001*
Total correct answers	0.500	<0.001*

* Significant p≤0,05;

r = correlation coefficient

According to multiple linear regression, increasing 1 point in total correct answers with a semantic cue reduces 0.399 points in total essential information remembered, in partial retelling, an increase of 1 point in the total number of incorrect answers with a phonemic cue reduces 0.248 points in the total of essential information remembered, in the partial retelling, 0.502 points in the total present information remembered in the partial retelling and 0.720 points in the total of ideas remembered in the full retelling, and an increase of 1 point in the total number of correct answers in the naming reduces 0.506 points in the total of ideas remembered in the full retelling.

### Performance in closed questions and associated factors

The result of the univariate analyzes between the total correct answers in the questions of the narrative discourse test and the explanatory variables are shown in tables 4 and 5. There was only moderate direct correlation between the total correct answers in the narrative discourse and the total number of responses correct in naming. A Multiple Linear Regression model was built, having as an outcome the performance in the questions of the narrative discourse. The backwards method was adopted, removing the variables with the highest p-value until the final adjusted model was obtained, however, no variable remained in the model.

**Table 4:** Results of the univariate analysis for the association between performance on the MAC Battery narrative discourse test questions and the variables gender, education, and oral language adequacy

Variable		Average (SD)	p value
Gender	**Female**	9.4 (3.0)	0.575
	**Male**	9.8 (2.9)	
School year	**6th**	9.3 (3.1)	0.319
	**7th**	9.0 (2.9)	
	**8th**	10.1 (3.0)	
	**9th**	10.5 (1.4)	
Ooral language	**Adequate**	9.6 (3.1)	0.911
	**Inappropriate**	9.6 (2.7)	

SD = standard deviation

**Table 5:** Pearson's correlation results for the association between performance of the MAC Battery narrative discourse test questions and the variables, age, memory, oral language, and picture naming

Variable	r	p value
Age	0.188	0.061
Memory	0.060	0.561
Working memory	0.057	0.568
Digit ascending ordering	0.072	0.485
Auditory span of words into sentences	0.064	0.537
Episodic-semantic verbal memory	0.062	0.562
Immediate recall	0.110	0.287
Late recall	0.088	0.393
Recognition	-0.057	0.582
Long-term semantic memory	-0.010	0.920
Short term visual memory	-0.034	0.742
Prospective memory	-0.108	0.294
Oral language	-0.097	0.345
Total correct answers (naming)	0.441	<0.001*

* Significant p≤0,05;

r = correlation coefficient

## DISCUSSION

This study verified the production and understanding of oral narrative discourse and associated factors of adolescents aged from 11 to 16 years of age from the 6th to 9th grades of public elementary schools. Picture naming was the only variable that predicted performance in oral narrative discourse tasks (partial and full retelling and answers to closed questions). In a qualitative analysis the adolescents were able to recount the story with adequate organization and planning in both a coherent and fluent manner. The performance was similar to that of young adults with a lower educational level (two to seven years of schooling)^(17)^. Most were able to infer the moral of the story and there was no difference in the performance of adolescents considering education levels and gender.

Vocabulary refers to the breadth and lexical diversity that one possess in relation to a language. It refers to the ability to understand terms and use them to acquire and convey meaning^(24)^. Naming, on the other hand, is an important skill for vocabulary building and involves a series of relatively distinct but interacting mental representations and cognitive processes, such as recognition of the visual stimulus as a familiar concept, access to the meaning of the visualized item, access to phonological structuring, and motor programming for oral production. Vocabulary and naming are closely related skills, as they mutually contribute to one another development and involve lexical access capacity and the quality of semantic representation in the lexicon^(24,25)^. In general terms in this study, the adolescents who performed better in naming also performed better in retelling tasks (partial and complete). To express the content of a story, several linguistic skills interact^(6)^, such as phonology, morphology, lexicon and grammatical processing^(19)^. In research carried out with children, vocabulary and naming were identified as a significant predictor of oral discourse score. Thus, the results corroborate other studies, as among the skills that contribute to oral narrative we have naming, and for the expression of the narrative discourse, the ability of lexical and preserved access is necessary, being directly proportional skills^(11)^.

The results of this study showed that the greater the difficulty in oral narrative discourse, the greater the difficulty in naming pictures, even after semantic and phonemic assistance. The total number of correct answers with semantic cues is inversely proportional to the total of essential information remembered in the partial retelling. Increasing the score of the total number of incorrect answers with phonemic cues reduces the score on the total of information (essential and present) and on the total of ideas remembered in the complete retelling. Another study found that participants with a higher educational level performed better in naming with assistance and that phonemic cues benefited participants older than eight years of age in formal education. Studies show that less educated participants do not benefit from assistance to retrieve a name, demonstrating a lack of knowledge of the lexicon^(26)^.

Regarding the production of oral narrative discourse studies state that to do this in a coherent way, the ability to retrieve information is required^(27)^ and this evolves as reading ability progresses^(16)^. Environmental stimuli are also essential in formulating a coherent discourse^(15)^. This study suggests that the difficulty in naming pictures with semantic and phonemic cues can be attributed to 1. lack of lexical knowledge, 2. graduation from the naming test with criteria of difficulty in another language, 3. lacking environmental stimulation, 4. reduction in reading habits, 5. level of education of adolescents, 6. restricted semantic memory and 7. infrequent words for adolescents.

During adolescence, memory development, and progress in deductive reasoning takes place^(1)^. For the retelling of a story, working memory is essential to maintain the theme, coherence and to retrieve information^(12)^. A study carried out with children verified the association between working memory and oral narrative skills^(28)^ and the direct contribution of memory to the understanding of oral narrative^(14)^. In the present study, there was no effect of memory in relation to the tasks of comprehension and production of narrative discourse in adolescents. The test used consisted of a short text, with only five short paragraphs and sought to assess the storage capacity of linguistic material. The MAC Battery test was designed for neurological patients, and the NEUPSILIN test aims to investigate the individual's neuropsychological performance in a simple and quick manner, in addition it does little to assess semantic memory and one can consider the possibility that the level of the tasks was easy for typically developed adolescents. Further studies are suggested to confirm this observation.

There was no influence of school year on the performance of either the partial or complete retelling, nor on the number of correct answers to the comprehension questions of the narrative discourse of the adolescents studied, however studies have shown that the influence of the school year is more significant in childhood. A Brazilian study evaluated the comprehension and production of oral narration of children aged from five to eleven years of age, showing that older children answered more questions correctly than younger children^(14)^. In another Brazilian study, carried out with children aged from six to twelve years of age, there were preliminary signs of a gradual improvement as children got older^(29)^. It is worth noting that these studies compared the results between children and adolescents, covering a greater age difference. This study only tested adolescents and this factor may have led to a difference in the results compared to other studies that evaluated children and adolescents. Future studies comparing high school adolescents may show an influence of age and education.

Oral discourse is a complex activity that requires a network of language skills and adolescents must structure and process events linked through logical relationships. The elaboration of a spontaneous story demands more cognitive resources than the retelling after listening to the text^(6)^. This study didn’t find a relationship between the general oral language score measured through the NEUPSILIN test tasks and the MAC Battery narrative discourse test score. It should be considered that, in general, the sample was not very heterogeneous in linguistic terms, as adolescents with alterations in language development were not included, consequently no associations were found between oral language and the test of narrative discourse. The associations may appear if we had of compared individuals with typical development and individuals with oral and written language disorders. Furthermore, the MAC Battery's narrative discourse task does not assess spontaneous discourse and only uses a narrative context^(17)^. This data points to the need to think about and implement oral language assessments that present tasks of greater linguistic complexity. It also indicates the possibility of standardizing the assessment of spontaneous speech and analyzing the association of the general oral language score in a neuropsychological test and the relationship of oral narrative discourse in adolescents with typical development and in those with language disorders. Therefore, we suggested evaluating oral narrative through different tasks and contexts, such as the performance of spontaneous narrative discourse, without pictures, through a previously stipulated theme, according to life experience, using the same analysis criteria of the MAC Battery.

There was no statistically significant correlation between gender in all tasks of the oral narrative discourse test, which corroborates other studies. A study that characterized the discursive aspects of adolescents in the 6th year of elementary school found a similar performance between boys and girls in both oral and written language^(8)^. In another study carried out with typical development adolescents, there were no statistically significant difference between genders in the performance of oral narrative^(18)^.

Research shows that linguistic competence in adolescence is similar to that of adults^(17)^, with an evolution of understanding^(2)^, increased information processing and understanding^(1)^. In this study when comparing the reference values of the MAC Battery, the adolescents performed close to that of young adults aged between 19-39 years of age in all oral narrative discourse tasks (partial retelling, full retelling and text comprehension^(17)^.

Of the six types of deviant communicative behavior in the narrative discourse, the adolescents presented non-timeline of events (21% of the adolescents), added incorrect information (15%), inaccurate references (6%) and inaccurate lexicon (5%). In a qualitative analysis, the adolescents demonstrated deviant communicative behavior during the verbal production of the narrative discourse, such as: non-timeline of events, addition of incorrect information, imprecise references, and imprecise lexicon, but the percentages were all quite low. Research demonstrates that individuals with brain trauma, compared with a control group of patients without neurological damage, produce narratives with a greater number of errors in cohesion, coherence, vague speech, with errors in the organization of the linguistic micro and macrostructures^(10)^. The presence of deviant communicative behavior in adolescents can be indicative of language alterations resulting from neurofunctional disorders.

The presence of deviant communicative behavior appears to be more common in the production of narrative discourse by individuals with brain damage and not in adolescents with typical language development. The presence of such behavior can be clinically indicative of language alterations, resulting from neurofunctional failures in the right hemisphere, and should be identified by clinical speech, language and hearing therapists in the evaluation of adolescents with language disorders.

A study was carried out with adolescents from public schools with the object of contributing to teaching practices, aimed at building inferences by adolescents between 12 and 19 years of age from elementary school. The results indicated that students had difficulty in making inferences, even reading simple texts, however this situation changed significantly during the course of the workshops^(28)^. In the studied sample, 64% of the adolescents created a title for the story, but not related to the moral of the story. However according to the test application rules adolescents were free to choose a title for the story, which did not necessarily have to be morally related^(17)^. Most adolescents (77%) kept the original title and chose not to create an additional title. This result is in accordance with other studies and may indicate that the ability to make inferences from a text may be under development in this age group.

The studied sample processed the inference only after listening to the text. In another study carried out with children from public and private schools, it was found that the groups benefited from the complete retelling, i.e., after the second listening^(29)^. Processing is faster by adolescents with a greater development than in relation to children.

In the process of adapting the MAC Battery narrative discourse to children, the percentage of inference processing in the public school was 46.7% and 75% in the private school^(29)^. In a study carried out with adults with two to seven years of schooling the percentage of inference processing was 76% and those with more than eight years of schooling was 96%^(17)^. Adolescents showed an intermediate performance between children from private schools and adults with more than eight years of schooling, with 77% of the inference processing, demonstrated through the understanding of the text's moral. Regarding the ability to make inferences when comparing adolescents with children from public schools and with adults, an evolution can be found, reinforcing the need for validated assessment instruments at this stage. This result corroborates other research that describes the adolescent in the transitional stage between childhood and adulthood^(1)^, with evolution of deductive reasoning and figurative and abstract language^(1)^. Other research has also shown that the comprehension of narrative in adolescents has not yet been completed and is in the process of maturation of the brain^(30)^. Research describes that the use of language by adolescents is more refined compared to that used by children^(1)^, adults on the other hand are more capable of making inferences than adolescents, because they have more developed worldly knowledge^(2)^.

A limitation of this study was that it only presented data from adolescents from public schools and both national and international surveys show an association between linguistic performance and socioeconomic level^(21,29)^. Consequently, if the narrative discourse tests were carried out with adolescents from private schools this may well generate different results.

The task of the MAC Battery's narrative discourse seems adequate, even without any adaptations made for children^(2)^. Future studies should verify the performance of students from private schools as well as adolescents from 15 to 17 years of age. It is also suggested that future studies of the analysis of spontaneous narrative should consider the effect of socioeconomic conditions, culture and age on oral narrative discourse skills.

This study evaluated the skills of oral narrative speech of adolescents with typical development. The study was an exploratory one because no other studies were found that researched the language skills of Brazilian Portuguese speaking adolescents.

## CONCLUSION

Picture naming was the only variable predicting performance in the oral narrative discourse tasks, and adolescents who performed better in naming also performed better in retelling tasks.

There was no statistically significant effect between narrative discourse and school year, age, memory, and overall oral language score. In general, adolescents showed a low percentage of deviant communicative behavior during the full retelling of the story in the MAC Battery, indicating that in this age group there is a decrease in the frequency of disruptions in the production of speech with the ability to recount the story in a coherent and fluent way.

In the studied sample, the participants were able to understand and elaborate an oral narrative discourse in a similar way to adults with a lower educational level (from two to seven years) and the MAC Battery narrative discourse test can be used to assess adolescents without requiring any form of adaptation.

Author contributions: SGA participated in the study design, data collection and analysis, and manuscript writing; EABC participated in the study design, overall supervision and review of the manuscript; LMA participated in the study design and review of the manuscript; VOMR participated in the study design, overall supervision and review of the manuscript.
