# Supplementary material for: Performance of adolescents in oral narrative discourse and associated factors
Source: Codas. 2022 Apr 15;34(5):e20210207. doi: 10.1590/2317-1782/20212021207 (PMC9886174; doi:10.1590/2317-1782/20212021207)
Supplement: Translation

# Translation: Desempenho de adolescentes no discurso narrativo oral e fatores associados

**Authors:** Samantha Gomes Araújo, Vanessa de Oliveira Martins-Reis, Erica de Araújo Brandão Couto, Luciana Mendonça Alves

**Affiliations:** 1 Universidade Federal de Minas Gerais – UFMG - Belo Horizonte (MG), Brasil.; 2 Universidade de Brasília – UnB - Brasília (DF), Brasil.

**Keywords:** Discurso Narrativo, Adolescente, Testes de Linguagem, Desenvolvimento da Linguagem, Nomeação de Figuras

## Abstract

**Método:**

100 adolescentes de ambos os sexos, cursando do sexto ao nono ano do ensino fundamental, sem queixa de distúrbio de linguagem e aprendizagem foram submetidos à avaliação do discurso narrativo oral (Bateria MAC), nomeação de figuras por confrontação visual (Teste de Nomeação de Boston), provas de linguagem oral e memória (NEUPSILIN). Considerou-se como variável resposta o desempenho no discurso narrativo (reconto parcial e integral e resposta a questões fechadas). Após análises univariadas, foram construídos modelos de Regressão Linear Múltipla.

**Resultados:**

Apenas o desempenho geral na tarefa de nomeação foi considerado preditor do desempenho no discurso narrativo oral. Verificou-se associação diretamente proporcional entre as variáveis do discurso narrativo e da nomeação de figuras. A caracterização do desempenho dos adolescentes no reconto parcial e integral e na resposta às questões fechadas por idade, ano escolar e sexo será apresentada.

**Conclusão:**

Na amostra estudada, os indivíduos foram capazes de compreender e de elaborar um discurso narrativo oral de forma semelhante a adultos com menor nível de escolaridade (de dois a sete anos). Dessa forma, a prova de discurso narrativo da Bateria MAC pode ser utilizada para a avaliação de adolescentes sem adaptações.

## INTRODUÇÃO

A adolescência é considerada como a transição no desenvolvimento entre a infância e a idade adulta, abrangendo dos onze aos vinte anos de idade. A competência linguística nesta faixa etária é semelhante à do adulto^(1)^, com evolução da linguagem abstrata, da compreensão, do uso da linguagem^(2)^. O crescimento linguístico ocorre durante a infância, adolescência e na idade adulta, não tendo um ponto óbvio de conclusão, sendo, portanto, um sistema dinâmico e em constante ampliação^(3)^.

Uma das habilidades da linguagem oral é o discurso narrativo, que se define como a ordenação linguística de eventos e ações relacionados temporalmente^(4)^. Esta habilidade é fundamental para o desenvolvimento da narrativa escrita^(5)^, para o bem estar socioemocional e desempenho escolar^(6)^. Dessa forma, o discurso narrativo oral é uma habilidade linguística essencial na capacidade comunicativa durante a adolescência. No uso social e acadêmico, o discurso narrativo deve ser competente e requer a capacidade de lembrar eventos, organizar informações, entender as perspectivas epistemológicas e emocionais dos outros, empregar sintaxe complexa e vocabulário apropriado para expressar-se com clareza e precisão^(7)^.

Observa-se tanto na prática clínica quanto na literatura específica, uma limitação de conhecimentos sobre as habilidades linguísticas e cognitivas de adolescentes, principalmente falantes do português brasileiro. Consequentemente, os instrumentos de avaliação e estratégias de intervenção são escassos^(8)^. A falta de testes padronizados com versões devidamente adaptadas e normatizadas à população adolescente limita a obtenção de dados objetivos na avaliação da linguagem nessa faixa etária.

Um recurso encontrado para suprir a quantidade limitada de instrumentos de avaliação destinada ao público adolescente é adaptar testes validados e reconhecidos para a população de adultos, os quais tenham como objetivos a avaliação das funções linguísticas e cognitivas. Com relação ao processamento do discursivo narrativo oral em adolescentes falantes do português brasileiro, não foram localizados instrumentos padronizados para tal finalidade clínica.

### Caracterização do discurso narrativo oral

A estrutura global da narrativa geralmente é caracterizada por uma configuração inicial, ações complicadoras e resolução, que são hierarquicamente organizadas em torno de três segmentos: começo, meio e fim^(9)^.

A capacidade narrativa oral pode ser avaliada em duas modalidades: compreensão e produção. É composta por níveis de macroestrutura e microestrutura. A macroestrutura relaciona-se aos componentes entre as sentenças (coesão, coerência, formulação de um modelo mental ou a essência de uma história) enquanto a microestrutura refere-se à construção dentro da frase (aspectos semânticos, lexicais, fonológicos e sintáticos da emissão)^(10)^.

Uma pesquisa realizada com crianças bilíngues do primeiro e segundo ano escolar teve como objetivo investigar as relações entre os domínios de nível micro e macro de narrativas orais dentro e entre as línguas inglesa e espanhola por meio do reconto de histórias. Os resultados evidenciaram que padrões de desenvolvimento da narrativa oral podem ser semelhantes tanto na língua inglesa como na espanhola e uma série de correlações significativas dentro e entre línguas identificaram o vocabulário como um preditor significativo de pontuação do discurso de nível macro em ambas as línguas^(11)^.

Com relação à compreensão da narrativa oral, a habilidade de inferir uma informação que não é indicada diretamente na história é influenciada pelas características do texto, por fatores cognitivos e contextuais^(12)^. A literatura sugere que a habilidade de fazer inferência contribui para compreensão da linguagem^(13)^. As perguntas para avaliação da compreensão narrativa são de caráter literal, inferencial e visam obter informações sobre a capacidade de se compreender as sentenças, bem como realizar relações entre ideias centrais ao tema da narrativa^(14)^.

Já a prosódica é uma habilidade relevante para o processamento da estrutura discursiva, pois interage e adiciona valor a outros subsistemas de linguagem, como sintaxe e semântica, facilitando a compreensão da linguagem. As pistas prosódicas ajudam a segmentar o fluxo de fala em frases, palavras e sílabas, informam a estrutura sintática e enfatizam informações facilitando a compreensão do discurso narrativo^(15)^. Estudo realizado com 79 alunos da segunda a quinta série, na faixa etária de 7 a 12 anos, investigou os aspectos prosódicos na compreensão da linguagem oral e da leitura; sugere-se que idade e escolaridade estariam relacionadas à capacidade de compreensão da escuta. Crianças mais novas e com menor nível de escolaridade seriam mais dependentes dos aspectos prosódicos, enquanto crianças mais velhas e com maior escolaridade apresentariam habilidades cognitivas mais desenvolvidas, o que as capacitaria a compreender um texto independente da variação prosódica^(16)^.

### Discurso narrativo oral na adolescência

O discurso narrativo oral reflete o nível de desenvolvimento cognitivo^(3)^. Fruto do processamento linguístico e mnemônico entre outras funções cognitivas subjacentes^(17)^, é uma habilidade determinante na vida escolar e social do adolescente^(3)^. Os adolescentes podem demonstrar grande diversidade no uso de sintaxe complexa no discurso narrativo oral^(18)^.

A narrativa oral, com seus elementos constituintes, é aprendida de forma gradual, à medida que ocorre a maturação do Sistema Nervoso Central^(19)^, a progressão da leitura. Níveis de escolaridade^(20)^ e antecedentes socioeconômicos estão associados à habilidade linguística na adolescência^(21)^.

Em adolescentes com transtorno na linguagem, a organização mental da informação não é adequada, observando-se problemas na sequencialização e estruturação do discurso, que compromete a capacidade de narrar eventos^(2)^. Problemas de linguagem na adolescência constituem uma área de interesse para profissionais da área da saúde e da educação e impactam de forma negativa no desempenho acadêmico, na participação na esfera social e na orientação profissional. Contudo, para caracterizar os problemas de linguagem do adolescente, é necessário conhecer melhor sobre o desenvolvimento típico das habilidades linguísticas nessa faixa etária.

Realizado em adolescentes com desenvolvimento típico da linguagem, com idade média de 14 anos, outro estudo tinha por objetivo criar uma nova tarefa narrativa e procurar determinar se provocaria maior complexidade sintática do que uma tarefa conversacional. Esta pesquisa abordou a necessidade de ferramentas apropriadas para avaliar a capacidade da narrativa oral com complexidade sintática de adolescentes estudantes da oitava série. Os resultados verificaram que não houve diferenças estatística significativa no desempenho da narrativa oral entre meninos e meninas e a tarefa narrativa suscitou maior complexidade sintática do que a tarefa conversacional^(18)^.

A aplicação de avaliações de linguagem padronizadas possibilita mensurar as características linguísticas, auxilia na compreensão do processo de aquisição, contribui para o desenvolvimento das capacidades cognitivas, e é base para a intervenção fonoaudiológica. Estuda-se a linguagem de crianças e adultos, mas a transição, ou seja, a adolescência, ainda é pouco explorada, principalmente as habilidades do discurso narrativo oral. Compreender melhor o discurso narrativo oral na adolescência pode guiar o desenvolvimento de ferramentas eficazes para programas de intervenção nessa faixa etária^(5)^. Assim, o objetivo do presente estudo foi caracterizar o desempenho de adolescentes, do 6º ao 9º ano do ensino fundamental, na produção e compreensão do discurso narrativo oral, bem como a influência do sexo, ano escolar, idade, nomeação de figuras, escore geral de linguagem oral e memória. Além disso, buscou-se verificar se a tarefa do discurso narrativo oral da Bateria Montreal de Avaliação da Comunicação – Bateria MAC, validada e normatizada para adultos falantes do português brasileiro, pode ser usada para a população adolescente sem adaptações.

## MÉTODO

Trata-se de estudo observacional analítico transversal, aprovado pelo Comitê de Ética em Pesquisa da Universidade Federal de Minas Gerais sob parecer nº1.722.230.

Os adolescentes elegíveis a participação no estudo e seus pais, foram esclarecidos quanto aos aspectos voluntários do estudo, seus benefícios, suas etapas e assinaram o Termo de Consentimento Livre e Esclarecido (TCLE) – pais e responsáveis) e Termo de Assentimento Livre Esclarecido (TALE).

Foram incluídos 100 adolescentes de duas escolas públicas da mesma região da cidade de Belo Horizonte. Os participantes não possuíam diagnósticos de transtornos de aprendizagem, alterações sensoriais, neurológicas, cognitivas ou comportamentais, de acordo com relatos dos coordenadores das escolas, e eram falantes nativos do português brasileiro, sem fluência em outro idioma. As escolas já possuíam uma lista com os adolescentes com Transtornos do Neurodesenvolvimento e a partir desta foi possível realizar a exclusão prévia de alguns participantes. Os adolescentes referidos pelos professores e coordenadores com baixo desempenho escolar também não foram incluídos.

A faixa etária dos participantes variou de 11 a 16 anos de idade, com média de 12,9 anos (DP=1,2). Dos participantes 62% eram do gênero feminino. No período da coleta, os adolescentes cursavam o ensino fundamental nos seguintes anos escolares: 6º ano (n=34), 7º ano (n=23), 8º ano (n=25) e 9º ano (n=18).

Os instrumentos utilizados para a realização do estudo foram: prova do discurso narrativo oral da Bateria Montreal de Avaliação da Comunicação - Bateria MAC^(17)^, Teste de Nomeação de Boston-TNB^(22)^ e testes de linguagem oral e memória do Instrumento de Avaliação Neuropsicológica Breve (NEUPSILIN)^(23)^.

Para avaliação do discurso narrativo oral foi utilizada a tarefa da Bateria MAC, composta por reconto parcial da história, parágrafo por parágrafo, reconto integral da história, tarefa de dar título e observação de processamento de inferência (compreensão da moral da história)^(17)^. Para aplicação e análise, foram utilizados os procedimentos propostos pelos autores do teste no manual de instruções. Nas tarefas de reconto, os adolescentes ouviram um texto curto e recontaram de modo resumido e com as próprias palavras o que acabara de acontecer na história, primeiro a cada parágrafo (reconto parcial). Registrou-se as caselas correspondentes ao total de informações essenciais lembradas (pontuação máxima=18) e total de informações presentes lembradas (pontuação máxima=29). Em seguida, o adolescente escutava a mesma história e solicitou-se o reconto do mesmo texto por completo (reconto integral, pontuação máxima=13). Foram anotados os comportamentos comunicativos desviantes, como: observações pessoais abundantes, discurso tangencial, não-respeito à cronologia dos eventos, omissão de marcadores de relação, léxico impreciso, referências imprecisas, acréscimo de informações erradas e falta de fluência. Em seguida, o adolescente respondia oralmente a 12 questões sobre a história e, depois, oferecia-se a opção de dar um novo título para a história. Baseando-se nas tarefas de reconto, respostas às perguntas de compreensão, e na linguagem não verbal (como risos e comentários pessoais), verificou-se se a inferência da narrativa foi feita, ou seja, a compreensão da moral da história^(17)^. O tempo médio de aplicação da tarefa do Discurso Narrativo Oral foi de 15 minutos.

Para as tarefas de reconto da história, padronizou-se a leitura para que não houvesse diferença de entonação e prosódia que pudesse vir a interferir na compreensão do texto por parte do adolescente. Para isso, foi realizada gravação do texto do teste, por um ator com 12 anos de experiência em teatro, utilizando-se o gravador Sony WDC WD3200BEVT- 75ZCT2.

A habilidade de nomear figuras foi avaliada por meio do TNB. As figuras foram apresentadas na ordem do teste, permitindo-se vinte segundos para a resposta. Se o adolescente produziu uma resposta incorreta ou nenhuma resposta, o examinador ofereceu uma pista fonêmica. Se após essa pista o sujeito continuou produzindo uma resposta incorreta ou nenhuma resposta dentro de vinte segundos, uma sugestão semântica relacionada à imagem, já indicada na folha de resposta, foi fornecida. A transcrição das respostas dadas pelos sujeitos foi registrada no protocolo de registro do teste. Para análise do teste, foi realizado o escore correspondente às respostas do adolescente, sendo essas: total de resposta corretas sem pista, total de resposta com pista fonêmica (correta e incorreta), total de resposta com pista semântica (correta e incorreta), total de respostas corretas geral (evocação espontânea + evocação com pistas)^(22)^. O tempo médio de aplicação do Teste de Nomeação de Boston foi 20 minutos.

Analisou-se o escore total do subteste de linguagem oral do NEUPSILIN^(24)^, que apresenta valores normativos para a população falante do português brasileiro e com idade superior a 12 anos. O subteste de linguagem oral é constituído por tarefas de nomeação, repetição, linguagem automática, compreensão e processamento de inferências. Atribuiu-se um ponto nas respostas corretas e zero nas incorretas; para análise somou-se o escore geral correspondente à pontuação total do adolescente. O desempenho do adolescente foi classificado em adequado e inadequado, considerando-se a referência do teste. O desempenho dos adolescentes foi registrado na folha de resposta padrão do teste. O tempo médio de aplicação foi de 7 minutos.

Também foi aplicado o subteste de memória do NEUPSILIN^(23)^. Analisou-se cada prova do subteste de memória separadamente, composto por tarefas de: memória de trabalho, memória verbal episódico-semântico, memória semântica de longo prazo e memória visual de curto prazo. Para análise dos resultados, foi feita avaliação quantitativa das respostas dos adolescentes, correspondente a cada tarefa da prova. O desempenho do estudante foi classificado em adequado e inadequado de acordo com os dados padronizados no teste. O tempo médio de aplicação foi de 7 minutos.

A coleta de dados foi realizada entre agosto de 2016 e março de 2017. Os testes citados no presente artigo foram aplicados separadamente, totalizando três sessões com cada adolescente. Todas as avaliações foram realizadas na própria escola, de forma individual, no horário em que os alunos frequentavam as aulas, em uma sala destinada para essa função; seguiu-se as normas de aplicação e análises padronizadas pelos testes.

Iniciou-se com a avaliação global da linguagem oral e da memória por meio do NEUPSILIN^(23)^. Em seguida aplicou-se o TNB^(22)^ e a Bateria MAC^(17)^.

O banco de dados foi construído com auxílio do Excel e após a devida análise de consistência foram realizadas análises descritivas univariadas utilizando o software Statistical Package for the Social Sciences (SPSS) 19.0. Inicialmente foi aplicado o teste Kolmogorov-Smirnov para avaliar normalidade das variáveis numéricas contínuas e todas apresentaram distribuição normal. Efetuou-se análise descritiva por meio do cálculo das frequências e medidas de tendência central e de dispersão (média e desvio-padrão). Foram aplicados os testes t de Student Simples para comparação entre duas médias independentes; Anova para comparar três médias independentes; e Correlação de Pearson para relacionar duas variáveis numéricas contínuas.

Para as análises, adotou-se como variável resposta o desempenho dos adolescentes na compreensão e expressão do discurso oral e como variáveis explicativas: gênero, ano escolar, idade, total de respostas corretas no Teste de Nomeação de Boston e escore global da linguagem oral (nomeação, repetição, linguagem automática, compreensão, e processamento de inferência) e memória (memória de trabalho, verbal episódico-semântica e prospectiva) do teste NEUPSILIN.

Por fim, foram construídos modelos de regressão linear múltipla, tendo como desfecho o discurso narrativo (reconto parcial e integral), e como variáveis explicativas todas aquelas que apresentaram valor p menor que 0,20 na análise univariada. Adotou-se o método backwards, com remoção das variáveis com maior valor de p até se obter o modelo final ajustado.

## RESULTADOS

A seção de resultados será apresentada em tópicos. Inicialmente serão apresentados os resultados das análises descritivas, univariadas e multivariadas do reconto parcial e integral. No segundo tópico, serão apresentados os resultados da associação entre as variáveis da tarefa de reconto da Bateria MAC e as variáveis do Teste de Nomeação de Boston. Em seguida um tópico específico para o desempenho nas questões fechadas.

### Reconto parcial e integral e fatores associados

A Tabela 1 demonstra os resultados da estatística descritiva do Discurso Narrativo dos adolescentes. Com relação ao título, inicialmente, apenas 30% deram títulos que demonstram que a inferência foi feita. A maioria dos adolescentes (79%) conservou o título e realizou inferência da moral da história (77%). O momento da inferência que prevaleceu foi após a primeira escuta com 42%.

**Tabela 1:** Estatística descritiva: Discurso Narrativo de adolescentes

Variável		Frequência (n)	Prevalência (%)
Observações pessoais abundantes			
sim		2	2,0
não		98	98,0
Discurso Tangencial			
sim		2	2,0
não		98	98,0
Não respeito à cronologia dos eventos			
sim		21	21,0
não		79	79,0
Omissão de marcadores de relação			
sim		0	0,0
não		100	100,0
Léxico impreciso			
sim		5	5,0
não		95	95,0
Referências imprecisas			
sim		6	6,0
não		94	94,0
Acréscimo de informações erradas			
sim		15	15,0
não		85	85,0
Falta de fluência			
sim		1	1,0
não		99	99,0
Título dado pelo adolescente			
Título que demonstra que a inferência foi feita		30	30,0
Título com relação com a história, mas não indicativo do processamento da inferência.		64	64,0
Título sem qualquer relação com a história ou com a relação incorreta.		6	6,0
Aluno conservou o título			
sim		79	79,0
não		21	21,0
Pontuação para novo título			
Título que demonstra que a inferência foi feita.		15	15,0
Título com relação com a história, mas não indicativo do processamento da inferência.		6	6,0
Título sem qualquer relação com a história ou com a relação incorreta.		2	2,0
Conservou o título		77	77,0
Inferência feita			
sim		77	77,0
Não		23	23,0
Momento que a inferência foi processada			
0- não houve		24	24,0
1- Durante		2	2,0
2- após a primeira leitura		42	42,0
3- Durante		3	3,0
4- após a segunda leitura		24	24,0
5- no fornecimento do primeiro título		0	0,0
6 – nas questões de compreensão do texto		5	5,0
7 – no fornecimento do segundo título		0	0,0
8 – outro momento		0	0,0
Variável	**Total - teste**	**Média**	**DP**
Total de informações essenciais lembradas no reconto parcial	18	12,6	3,3
Total de informações presentes lembradas no reconto parcial	29	16,0	4,6
Total de ideias lembradas no reconto integral	13	9,8	2,5
Total de resposta corretas nas questões	12	9,6	3,0

DP = desvio padrão

Dos seis tipos de comportamentos comunicativos desviantes no discurso narrativo, os adolescentes apresentaram não cronologia dos eventos (21% dos adolescentes), acréscimo de informações erradas (15%), referências imprecisas (6%) e léxico impreciso (5%).

Em um total de 18 informações essenciais lembradas, a média de respostas dos adolescentes foi de 12,6 pontos e em 29 informações presentes lembradas, a média foi de 16 pontos, no reconto parcial da história. Com relação ao reconto integral da história, no total de 13 informações lembradas, a média de respostas dos adolescentes foi de 9,8 pontos. Já na avaliação da compreensão do texto, no total de 12 questões a média foi de 9,6 pontos (Tabela 1).

Neste estudo não se observa associação entre o desempenho dos adolescentes nos recontos da prova de discurso narrativo e as variáveis explicativas sexo, escolaridade e adequação na linguagem oral, segundo os resultados das análises univariadas entre o desempenho na prova de discurso e as variáveis explicativas.

A Tabela 2 apresenta os resultados da correlação de Pearson entre o desempenho no reconto parcial e integral da Bateria MAC e as variáveis idade, memória, desempenho geral em linguagem oral e nomeação de figuras. Observa-se correlação moderada direta entre o reconto parcial e integral e o total de respostas corretas na nomeação. Nesse caso, quanto melhor o desempenho na nomeação de figuras do Teste de Nomeação de Boston, maior o total de informações essenciais e presentes lembradas no reconto parcial e o total de informações lembradas no reconto integral. As demais variáveis não apresentaram associação com o reconto parcial e integral.

**Tabela 2:** Resultados da Correlação de Pearson para associação entre o desempenho no reconto da prova de discurso narrativo da Bateria MAC e as variáveis idade, memória, linguagem oral e nomeação de figuras

**Variável**	**Total de informações essenciais lembradas no reconto parcial**		**Total de informações presentes lembradas no reconto parcial**		**Total de ideias lembradas no reconto integral**	
	**r**	**valor p**	**r**	**valor p**	**r**	**valor p**
**Idade**	0,126	0,212	0,122	0,227	0,128	0,206
**Memória**	0,142	0,166	0,160	0,120	0,024	0,817
**Memória de trabalho**	0,101	0,326	0,122	0,238	-0,051	0,619
**Ordenamento ascendente de dígitos**	0,051	0,625	0,132	0,200	-0,029	0,777
**Span auditivo de palavras em sentenças**	0,122	0,277	0,121	0,242	-0,021	0,841
**Memória verbal episódico-semântica**	0,129	0,209	0,104	0,312	0,108	0,293
**Evocação Imediata**	0,072	0,485	0,030	0,771	-0,005	0,962
**Evocação Tardia**	0,179	0,082	0,161	0,118	0,122	0,082
**Reconhecimento**	0,043	0,679	0,040	0,700	0,114	0,269
**Memória semântica de longo prazo**	0,010	0,922	0,075	0,495	0,034	0,746
**Memória visual de curto prazo**	-0,020	0,893	0,072	0,486	-0,004	0,965
**Linguagem Oral**	-0,041	0,692	0,047	0,650	-0,083	0,424
**Total de respostas corretas (nomeação)**	0,443	<0,001*	0,463	<0,001*	0,500	<0,001*

* p<0,05;

r = coeficiente de correlação

O desempenho no Teste de Nomeação de Boston permaneceu nos três modelos de regressão linear múltipla. De acordo com os modelos finais: o aumento de 1 ponto no total de respostas corretas na nomeação aumenta 0,322 pontos no total de informações essenciais lembradas no reconto parcial; 0,225 ponto no total de informações presentes lembradas no reconto parcial; e 0,192 ponto no total de ideias lembradas no reconto integral.

### Discurso narrativo versus nomeação de figuras

Como o desempenho geral no Teste de Nomeação de Boston foi a única variável preditora do desempenho dos adolescentes no reconto, optou-se por verificar a associação de todas as variáveis do teste com o discurso narrativo. Os resultados da Correlação de Pearson são apresentados na Tabela 3.

**Tabela 3:** Correlações de Pearson entre o desempenho no reconto parcial e integral e o desempenho na nomeação com e sem pistas semânticas e fonêmicas

Total de informações essenciais lembradas no reconto parcial	r	valor p
Total de respostas corretas sem pista	0,461	<0,001*
Total de respostas corretas com pista semântica	-0,225	0,033*
Total de resposta incorreta com pista semântica	-0,400	<0,001*
Total de resposta correta com pista fonêmica	0,068	0,204
Total de resposta incorreta com pista fonêmica	-0,479	<0,001*
Total de respostas corretas	0,443	<0,001*
Total de informações presentes lembradas: reconto parcial	**r**	**valor p**
Total de respostas corretas sem pista	0,468	<0,001*
Total de respostas corretas com pista semântica	-0,201	0,046*
Total de resposta incorreta com pista semântica	-0,394	<0,001*
Total de resposta correta com pista fonêmica	0,092	0,364
Total de resposta incorreta com pista fonêmica	-0,502	<0,001*
Total de respostas corretas	0,463	<0,001*
Total de ideias lembradas: reconto integral	**r**	**valor p**
Total de respostas corretas sem pista	0,556	<0,001*
Total de respostas corretas com pista semântica	-0,280	0,005*
Total de resposta incorreta com pista semântica	-0,495	<0,001*
Total de resposta correta com pista fonêmica	0,018	0,856
Total de resposta incorreta com pista fonêmica	-0,559	<0,001*
Total de respostas corretas	0,500	<0,001*

* p<0,05;

r = coeficiente de correlação

De acordo com a regressão linear múltipla: o aumento de 1 ponto no total de respostas corretas com pista semântica reduz 0,399 ponto no total de informações essenciais lembradas no reconto parcial; o aumento de 1 ponto no total de respostas incorretas com pista fonêmica, reduz 0,248 ponto no total de informações essenciais lembradas no reconto parcial, 0,502 ponto no total de informações presentes lembradas no reconto parcial e 0,720 ponto no total de ideias lembradas no reconto integral; e o aumento de 1 ponto no total de respostas corretas na nomeação reduz 0,506 ponto no total de ideias lembradas no reconto integral.

### Desempenho nas questões fechadas e fatores associados

Os resultados das análises univariadas entre o total de acertos nas questões da prova de discurso narrativo e as variáveis explicativas são apresentados nas Tabelas 4 e 5. Observou-se apenas correlação moderada direta entre o total de respostas corretas no discurso narrativo e o total de respostas corretas na nomeação. Foi construído modelo de Regressão Linear Múltipla, tendo como desfecho o desempenho nas questões do discurso narrativo. Adotou-se o método backwards, com remoção das variáveis com maior valor de p até se obter o modelo final ajustado, entretanto, nenhuma variável permaneceu no modelo.

**Tabela 4:** Resultados da análise univariada para associação entre o desempenho nas questões da prova de discurso narrativo da Bateria MAC e as variáveis gênero, escolaridade e adequação na linguagem oral

Variável		Média (DP)	valor p
Gênero	Feminino	9,4 (3,0)	0,575
	Masculino	9,8 (2,9)	
Ano Escolar	6º	9,3 (3,1)	0,319
	7º	9,0 (2,9)	
	8º	10,1 (3,0)	
	9º	10,5 (1,4)	
Linguagem Oral	Adequado	9,6 (3,1)	0,911
	Inadequado	9,6 (2,7)	

DP = desvio padrão

**Tabela 5:** Resultados da Correlação de Pearson para associação entre o desempenho nas questões da prova de discurso narrativo da Bateria MAC e as variáveis idade, memória, linguagem oral e nomeação de figuras

Variável	r	valor p
Idade	0,188	0,061
Memória	0,060	0,561
Memória de trabalho	0,057	0,568
Ordenamento ascendente de dígitos	0,072	0,485
Span auditivo de palavras em sentenças	0,064	0,537
Memória verbal episódico-semântica	0,062	0,562
Evocação Imediata	0,110	0,287
Evocação Tardia	0,088	0,393
Reconhecimento	-0,057	0,582
Memória semântica de longo prazo	-0,010	0,920
Memória visual de curto prazo	-0,034	0,742
Memória prospectiva	-0,108	0,294
Linguagem Oral	-0,097	0,345
Total de respostas corretas (nomeação)	0,441	<0,001*

* p<0,05;

r = coeficiente de correlação

## DISCUSSÃO

O presente estudo verificou o desempenho de adolescentes de 11 a 16 anos, do 6º ao 9º ano do ensino fundamental, de escola pública, na produção e compreensão do discurso narrativo oral e os fatores associados. A nomeação de figuras foi à única variável preditora do desempenho nas tarefas do discurso narrativo oral (reconto parcial e integral e respostas às questões fechadas). Em análise qualitativa, os adolescentes foram capazes de recontar uma história com organização e planejamento adequados, de forma coerente e fluente. O desempenho foi semelhante ao de adultos jovens com menor escolaridade (dois a sete anos de estudo)^(17)^. A maioria foi capaz de inferir a moral da história e não houve diferença no desempenho dos adolescentes considerando os níveis de escolaridade e sexo.

O vocabulário diz respeito à amplitude e à diversidade lexical que se possui em relação a uma língua. Refere-se à capacidade de compreender os termos e utilizá-los para adquirir e transmitir significado^(24)^. Já a nomeação é uma habilidade importante para a construção do vocabulário e envolve uma série de representações mentais e processos cognitivos relativamente distintos, mas em interação, tais como: reconhecimento do estímulo visual como um conceito familiar, acesso ao significado do item visualizado, acesso à estruturação fonológica, e programação motora para a produção oral. O vocabulário e a nomeação são habilidades intimamente relacionadas, de forma que uma contribui para o desenvolvimento da outra mutuamente e envolvem a capacidade de acesso lexical e a qualidade da representação semântica no léxico^(24,25)^. Em linhas gerais, no presente estudo, os adolescentes que apresentaram melhor desempenho na nomeação também o apresentaram nas tarefas de reconto (parcial e integral). Para expressar o conteúdo de uma história, interagem várias habilidades linguísticas^(6)^, como a fonologia, morfologia, léxico e processamento gramatical^(19)^. Em pesquisa realizada com crianças, identificou-se o vocabulário e a nomeação como um preditor significativo de pontuação do discurso oral. Assim, os resultados corroboram a literatura, pois dentre as habilidades que contribuem para a narrativa oral temos a nomeação, e para expressão do discurso narrativo é necessária a capacidade do acesso lexical e preservado, sendo habilidades diretamente proporcionais^(11)^.

Os resultados do presente estudo demonstraram que quanto maior a dificuldade no discurso narrativo oral, maior a dificuldade na nomeação de figuras, mesmo após as facilitações semânticas e fonêmicas. O total de respostas corretas com pista semântica é inversamente proporcional ao total de informações essenciais lembradas no reconto parcial. O aumento da pontuação no total de respostas incorretas com pista fonêmica reduz a pontuação no total de informações (essenciais e presentes) e no total de ideias lembradas no reconto integral. Outro estudo verificou que indivíduos com nível educacional mais alto obtiveram melhor desempenho na nomeação com uso de facilitações e que pistas fonêmicas beneficiaram os indivíduos com mais de oito anos de instrução formal. A literatura aponta que indivíduos menos escolarizados não se beneficiam de facilitações para recuperação do nome, evidenciando a falta de conhecimento do léxico^(26)^.

Com relação à produção do discurso narrativo oral, a literatura descreve que para fazê-lo de forma coerente, é necessária a habilidade de recuperação das informações^(27)^ e esta evolui à medida que ocorre progressão da leitura^(16)^. Os estímulos ambientais também são essenciais na formulação de um discurso coerente^(15)^. O presente estudo sugere que a dificuldade na nomeação de figuras com pista semântica e fonêmica pode ser atribuída à: 1. falta do conhecimento lexical; 2. graduação do teste de nomeação com critérios de dificuldade de outra língua; 3. estimulação ambiental carente; 4. redução do hábito da leitura; 5. nível de escolaridade dos adolescentes; 6. memória semântica restrita; e 7. palavras pouco frequentes para adolescentes.

Na adolescência ocorre o desenvolvimento da memória e progresso no raciocínio dedutivo^(1)^. Para o reconto da história, a memória funcional é essencial para manter o tema, a coerência e para recuperar informações^(12)^. Estudo realizado com crianças verificou a associação entre memória de trabalho e habilidades da narrativa oral^(28)^ e a contribuição direta da memória para compreensão da narrativa oral^(14)^. No presente estudo não se observou efeito da memória com relação às tarefas de compreensão e produção do discurso narrativo nos adolescentes. A prova utilizada é composta por um texto curto, com apenas cinco pequenos parágrafos e busca avaliar a capacidade de armazenamento do material linguístico. A Bateria MAC foi delineada para pacientes neurológicos, e o teste NEUPSILIN tem como propósito investigar de forma simples e rápida o desempenho neuropsicológico do indivíduo, além disso, avalia pouco a memória semântica; pode-se considerar a possibilidade de as tarefas terem sido de nível fácil para adolescentes com desenvolvimento típico. Sugerem-se novos estudos para confirmação dessa observação.

Não foi observada, dentre os adolescentes estudados, influência do ano escolar no desempenho do reconto parcial e integral, nem nos acertos nas questões de compreensão do discurso narrativo. No entanto, estudos comprovam que a influência do ano escolar é mais significativa na infância. Estudo brasileiro avaliou a compreensão e a produção da narração oral de crianças de cinco a 11 anos e 11 meses, demostrando que as crianças mais velhas apresentaram mais acertos do que as crianças mais jovens^(14)^. Em outro estudo brasileiro, realizado com crianças de seis a doze anos, houve indícios preliminares de melhora gradual com o avanço da idade^(29)^. Vale ressaltar que estas pesquisas compararam os resultados entre crianças e adolescentes, abrangendo uma diferença maior de faixa etária. O presente estudo testou apenas adolescentes e este fator pode ter levado a diferença de resultados com relação as demais pesquisas que avaliaram crianças e adolescentes. Estudos futuros comparando com adolescentes do ensino médio poderão apontar o efeito de idade e escolaridade.

O discurso é uma atividade complexa que requer uma rede de habilidades linguísticas; o adolescente deve estruturar e processar eventos interligados através de relações lógicas. A elaboração de uma história espontânea demanda mais recursos cognitivos do que o reconto após a escuta do texto^(6)^. No presente estudo, não foi observada relação entre a pontuação geral da linguagem oral medida por meio das tarefas do teste NEUPSILIN e o escore da prova do discurso narrativo da Bateria MAC. Deve-se considerar que de maneira geral, a amostra não era muito heterogênea em termos linguísticos, pois não foram incluídos os adolescentes com alterações no desenvolvimento da linguagem, assim não foram encontradas associações entre a linguagem oral e a prova do discurso narrativo. A associação pode aparecer quando estivermos comparando indivíduos com desenvolvimento típico e indivíduos com transtornos da linguagem oral e escrita. Além disso, a tarefa do discurso narrativo da Bateria MAC não avalia o discurso espontâneo e utiliza apenas um contexto narrativo^(17)^. Este dado aponta para a necessidade de se pensar e implementar avaliações de linguagem oral que apresentem maior complexidade linguísticas nas tarefas. Indica também, a possibilidade de padronização de avaliação de fala espontânea e análise da associação do escore geral de linguagem oral em teste neuropsicológico e da relação do discurso narrativo oral em adolescentes com desenvolvimento típico e naqueles com transtorno de linguagem. Sendo assim, sugere-se a avaliação da narrativa oral por meio de tarefas e contextos diferenciados, como o desempenho do discurso narrativo espontâneo, sem figuras, mediante um tema previamente estipulado, de acordo com a experiência de vida, utilizando os mesmos critérios de análise da Bateria MAC.

Não se observou correlação estatisticamente significativa entre o sexo, em todas as tarefas do teste do discurso narrativo oral, o que corrobora a literatura; estudo que caracterizou os aspectos discursivos de adolescentes do 6º ano do ensino fundamental observou semelhança de desempenho entre meninos e meninas tanto na linguagem oral quanto na linguagem escrita^(8)^. Em outro estudo realizado com adolescentes com desenvolvimento típico, não houve diferença estatisticamente significativa entre os sexos no desempenho da narrativa oral^(18)^.

A literatura descreve que a competência linguística da adolescência é semelhante à do adulto^(17)^, ocorrendo a evolução da compreensão^(2)^, aumento do processamento da informação e do entendimento^(1)^. No presente estudo, quando comparado aos valores de referência da Bateria MAC, os adolescentes tiveram desempenho próximo ao de adultos jovens de 19-39 anos em todas as provas do discurso narrativo oral (reconto parcial, reconto integral e compreensão do texto^(17)^.

Dos seis tipos de comportamentos comunicativos desviantes no discurso narrativo, os adolescentes apresentaram não cronologia dos eventos (21% dos adolescentes), acréscimo de informações erradas (15%), referências imprecisas (6%) e léxico impreciso (5%).

Em análise qualitativa, os adolescentes apresentaram comportamentos comunicativos desviantes durante as produções verbais do discurso narrativo, como: não cronologia dos eventos, acréscimo de informações erradas, referências imprecisas, e léxico impreciso, porém todos estes em baixa porcentagem. A literatura demonstra que indivíduos com traumatismo cerebral, comparados com grupo controle de pacientes sem lesões neurológicas, produzem narrativas com maiores erros de coesão, coerência, discursos vagos, com erros na organização da micro e macroestrutura linguística^(10)^. Assim, a presença de comportamentos comunicativos desviantes em adolescentes pode ser indicativa de alterações linguísticas decorrentes de transtornos neurofuncionais.

Dessa forma, os comportamentos comunicativos desviantes parecem estar mais presentes na produção do discurso narrativo em indivíduos com lesão cerebral e não em adolescentes com desenvolvimento típico da linguagem. A presença de tais comportamentos pode ser indicativa clínica de alterações na linguagem, decorrentes de falhas neurofuncionais no hemisfério direito, devendo ser observada pelos fonoaudiólogos clínicos na avaliação de adolescentes com queixas de transtornos da linguagem.

Um estudo foi realizado com adolescentes de escola pública, com o objetivo de contribuir com as práticas docentes direcionadas à construção de inferências por parte de adolescentes do ensino fundamental com idades de 12 a 19 anos. Os resultados indicaram que os estudantes apresentaram dificuldade em fazer inferência, mesmo a partir da leitura de textos simples, no entanto, essa situação mudou significativamente no decorrer da aplicação das oficinas^(28)^.

Na amostra estudada, 64% dos adolescentes elaboraram título para a história, mas não relacionado a moral da história. No entanto, de acordo com as normas de aplicação do teste, os adolescentes podiam escolher livremente um título para a história e que não necessariamente deveria ter relação com a moral^(17)^. A maioria dos adolescentes (77%) conservou o título dado no primeiro momento, optando por não elaborar um segundo título. Assim, este resultado está de acordo com a literatura e pode indicar que habilidade de fazer inferências a partir de um texto, pode estar em desenvolvimento nessa faixa etária.

Na amostra estudada, a inferência foi processada após uma única escuta do texto. Em pesquisa realizada com crianças de escola pública e privada, constatou-se que os grupos se beneficiaram do reconto integral, ou seja, após a segunda escuta^(29)^. Assim, observa-se um processamento mais rápido por parte dos adolescentes e um desenvolvimento maior com relação as crianças.

No processo de adaptação do discurso narrativo da Bateria MAC para crianças, o percentual do processamento de inferências, na escola pública foi de 46,7% e na escola privada de 75%^(29)^. Em estudo realizado com adultos, o percentual do processamento da inferência, com escolaridade de dois a sete anos foi de 76% e com mais de oito anos de escolaridade foi de 96%^(17)^. Os adolescentes apresentaram um desempenho intermediário entre crianças de escola privada e adultos com mais de oito anos de estudo, com 77% do processamento da inferência, demonstrado por meio da compreensão da moral do texto. Assim, com relação à capacidade de fazer inferências, quando se comparam adolescentes com crianças de escola pública e com adultos, observa-se uma evolução, reforçando a necessidade de instrumentos de avaliação validados nesta fase. Este resultado corrobora a literatura que descreve o adolescente em processo de transição entre a infância e a fase adulta^(1)^, com evolução do raciocínio dedutivo e da linguagem figurada e abstrata^(1)^. Outra pesquisa demonstrou que a compreensão da narrativa em adolescente ainda não se completou e encontra-se em processo de maturação cerebral^(30)^. A literatura descreve que o uso da linguagem pelo adolescente é mais refinado em comparação ao uso pelas crianças^(1)^; já adultos possuem maior capacidade de fazer inferências, com relação aos adolescentes, por terem maior conhecimento de mundo^(2)^.

Uma limitação do estudo é apresentar apenas dados de adolescentes de escola pública, visto que pesquisas nacionais e internacionais apontam associação entre o desempenho linguístico e o nível socioeconômico^(21,29)^. Dessa forma, a aplicação de testes do discurso narrativo em adolescentes de escola privada poderia gerar resultados diferentes.

A prova do discurso narrativo da Bateria MAC parece adequada, mesmo sem as adaptações realizadas para o público infantil^(2)^. Estudos futuros deverão verificar o desempenho de estudantes de escola privada bem como o de adolescentes de 15 a 17 anos. Para estudos futuros, sugere-se a análise da narrativa espontânea e a verificação do efeito do nível socioeconômico, cultural, e de idade nas habilidades do discurso narrativo oral.

Assim, por meio desse estudo verificou-se as habilidades do discurso narrativo oral em adolescentes com desenvolvimento típico. Este estudo representa uma exploração inicial, visto que não foram encontrados estudos sobre essas habilidades linguísticas em populações compostas exclusivamente por adolescentes falantes do português brasileiro.

## CONCLUSÃO

A nomeação de figuras foi a única variável preditora do desempenho nas tarefas do discurso narrativo oral, sendo que os adolescentes que apresentaram melhor desempenho na nomeação, também o apresentaram nas tarefas de reconto.

Não se observou efeito estatisticamente significativo entre o discurso narrativo e o ano escolar, idade, memória e escore geral de linguagem oral. De maneira geral, os adolescentes apresentam baixa porcentagem de comportamentos comunicativos desviantes durante o reconto integral da história na Bateria MAC, indicando que nesta faixa etária ocorre a diminuição da frequência de rupturas na produção do discurso com capacidade de reconto da história de forma coerente e fluente.

Na amostra estudada, os indivíduos foram capazes de compreender e de elaborar um discurso narrativo oral de forma semelhante a adultos com menor nível de escolaridade (de dois a sete anos). Dessa forma, a prova de discurso narrativo da Bateria MAC pode ser utilizada para a avaliação de adolescentes sem adaptações.
